# Synthesis and Characterization of Cry2Ab–AVM Bioconjugate: Enhanced Affinity to Binding Proteins and Insecticidal Activity

**DOI:** 10.3390/toxins11090497

**Published:** 2019-08-27

**Authors:** Zhi-Zhen Pan, Lian Xu, Yi-Shu Zheng, Li-Yang Niu, Bo Liu, Nan-Yan Fu, Yan Shi, Qing-Xi Chen, Yu-Jing Zhu, Xiong Guan

**Affiliations:** 1 State Key Laboratory of Ecological Control of Fujian-Taiwan Crop Pests, Key Laboratory of Biopesticide and Chemical Biology of Ministry of Education, College of Plant Protection, Fujian Agriculture and Forestry University, Fuzhou 350002, China; 2 Agricultural Bio-Resources Research Institute, Fujian Academy of Agricultural Sciences, Fuzhou 350003, China; 3 School of Life Sciences, Xiamen University, Xiamen 361005, China; 4 MOE Key Laboratory for Analytical Science of Food Safety and Biology & Fujian Provincial Key Laboratory of Analysis and Detection Technology for Food Safety, College of Chemistry, Fuzhou University, Fuzhou 350116, China

**Keywords:** Cry2Ab–AVM bioconjugate, binding affinity, molecular docking

## Abstract

*Bacillus thuringiensis* insecticidal proteins (Bt toxins) have been widely used in crops for agricultural pest management and to reduce the use of chemical insecticides. Here, we have engineered Bt toxin Cry2Ab30 and bioconjugated it with 4”-O-succinyl avermectin (AVM) to synthesize Cry2Ab–AVM bioconjugate. It was found that Cry2Ab–AVM showed higher insecticidal activity against *Plutella xylostella*, up to 154.4 times compared to Cry2Ab30. The binding results showed that Cry2Ab–AVM binds to the cadherin-like binding protein fragments, the 10th and 11th cadherin repeat domains in the *P. xylostella* cadherin (PxCR_10–11_), with a much higher affinity (dissociation equilibrium constant *K_D_* = 3.44 nM) than Cry2Ab30 (*K_D_* = 28.7 nM). Molecular docking suggested that the macrolide lactone group of Cry2Ab–AVM ligand docking into the PxCR_10–11_ is a potential mechanism to enhance the binding affinity of Cry2Ab–AVM to PxCR_10–11_. These findings offer scope for the engineering of Bt toxins by bioconjugation for improved pest management.

## 1. Introduction

*Bacillus thuringiensis* insecticidal proteins (Bt toxins) have been shown to be highly effective in agricultural pest control [1,2]. Bt toxins are widely expressed in crops for agricultural pest management and reduce the use of chemical insecticides [3]. Bt toxins are considered as environmentally friendly biological pesticides as they can kill insect pests with little harm to most other organisms and cause few side effects to the ecosystem [4,5].

The interaction of Bt toxins with binding proteins on the surface of insect midgut cells can lead to oligomerization, membrane insertion, pore formation, and insect death [6,7,8,9,10]. The downregulation or deletion of these binding proteins may weaken or disrupt the interaction with Bt toxins, which would result in inefficient insect control or cause insect resistance [1,11,12,13]. Engineering of Bt toxin to enhance its affinity to binding proteins in insects can improve Bt toxin potency, expand Bt toxin specificity, and bypass receptor-related resistance mechanisms [14,15,16,17]. For example, Badram et al. developed a phage-assisted continuous evolution selection that created a rapidly evolving Bt toxin Cry1Ac which binds a cadherin-like receptor from the insect pest *Trichoplusia ni* that is not natively bound by wild-type Cry1Ac, with a high affinity to improve the insecticidal potency and overcome insect Bt toxin resistance [15].

In this study, we used bioconjugation to evolve the Bt toxin to enhance its insecticidal activity and improve affinity to its binding proteins. 1-Ethyl-3-(3-dimethylaminopropyl) carbodiimide (EDC) and *N*-hydroxysuccinimide (NHS) were selected as the couplers, which are carboxyl and amine-reactive zero-length crosslinkers [18]. EDC/NHS reacted with the carboxylic group of the bioconjugation ligand first, and formed an NHS ester intermediate that reacted with an amino group of Bt toxin to form a stable amide bond [19]. Cry2Ab30 was chosen as the target Bt toxin to be modified, which is a 65 kDa 3D-Cry of Bt toxin [20]. Cadherin, formed by a number of characteristic cadherin repeats (CR) domains, a membrane-proximal extracellular domain (MPED), a transmembrane domain, and a cytoplasm domain, serves as an important binding protein for several classes of Cry toxins [21]. For example, *Spodoptera exigua* cadherin was reported can bind both Cry1Ac and Cry2Aa [22]. Here, the 10th and 11th CR domains (1258–1602 aa) in the *Plutella xylostella* cadherin (PxCR_10–11_) were expressed and chosen as the Cry2Ab-binding protein [23]. Avermectin (AVM) belongs to a family of compounds called the macrocyclic lactones (MLs), and was introduced to the market in the 1980s as an antiparasitic drug and agricultural pesticide [24]. The mode of action of MLs is based on their interaction with the receptor channels for inhibitory neurotransmitters. However, MLs also irreversibly bind to other receptors with high affinity in a process involving macrocyclic groups, such as glutamate and glycine receptors [25,26]. Therefore, AVM was selected as the bioconjugation ligand with regarding to its structure–activity, with the aim of improving the affinity of Cry2Ab30 to its binding proteins. Here, AVM was first carbonylated to synthesize 4”-O-succinyl avermectin to improve its bioconjugation activity. Cry2Ab–AVM was then synthesized by bioconjugating 4”-O-succinyl avermectin onto Cry2Ab30 via EDC/NHS, which enhanced the insecticidal activity against *P. xylostella* up to 154.4-fold over Cry2Ab30 and led to binding of PxCR_10–11_ with a higher affinity. These results established an approach to the evolution of Bt toxins and provided a new platform for the engineering of other protein-binding biomolecules.

## 2. Results

### 2.1. Preparation and Characterization of Cry2Ab–AVM

**Figure 1:**
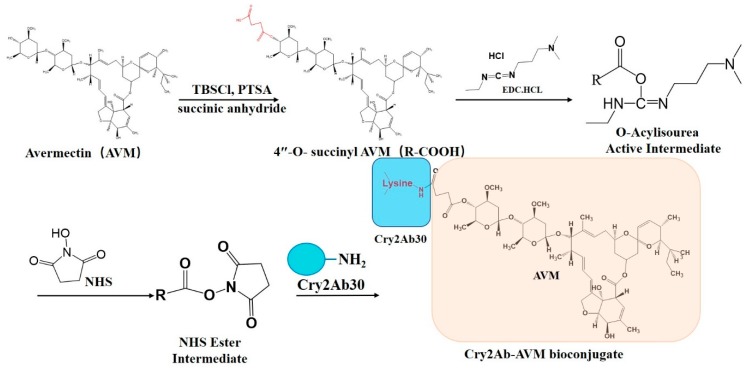
Schematic illustration of the procedures for bioconjugation of Cry2Ab–AVM.

To improve the insecticidal activity of Cry2Ab30, Cry2Ab–AVM was synthesized by bioconjugating 4”-O-succinyl avermectin onto Cry2Ab30 via EDC/NHS. In our previous study, we bioconjugated AVM onto Bt δ-endotoxin to form GSCS-BtA biocide, which had higher efficacy in reducing the abundance of some important crucifer pests while exhibiting low impact on their natural enemies [27]. Here, we made a modification on AVM to firstly improve its bioconjugation reactivity. AVM contains three hydroxyl groups, with the secondary allylic hydroxyl group at the 5 position is the most reactive, followed by the secondary hydroxyl group at the 4” position. The tertiary allylic hydroxyl group at the 7 position is too sterically hindered to be reactive [28]. The two reactive hydroxyl groups (5-OH and 4”-OH) have the potential to be modified by conjugation with Cry2Ab30. Considering that the 5-OH is located on the macrolide lactone group of AVM, which may interact with the binding proteins of Cry2Ab30, we selected the 4”-OH to be modified by conjugation with Cry2Ab30. 4”-O-succinyl avermectin was synthesized to create a carboxylic group on 4” position of AVM. The carboxylic group was then converted to NHS ester intermediate in the presence of EDC and NHS, followed by its reaction with amino groups on Cry2Ab30, thus forming a stable amide linkage (Figure 1). Excess EDC, NHS, 4”-O-succinyl avermectin, and NHS esters were removed using a PD-10 desalting column.

**Figure 2:**
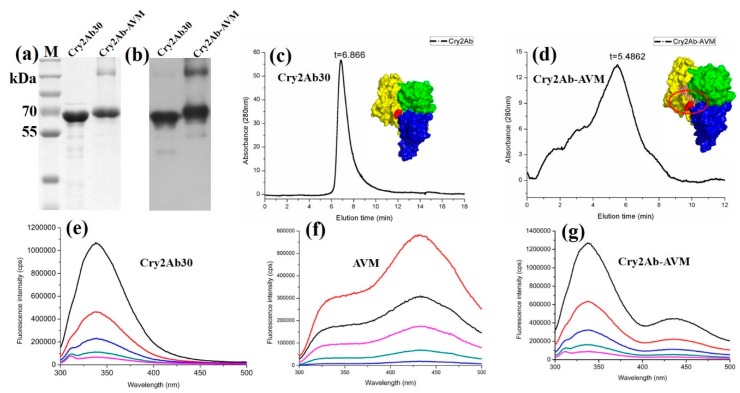
Characterization of Cry2Ab–AVM. Cry2Ab30 and Cry2Ab–AVM were evaluated by 10% SDS-PAGE (**a**) and Western blotting using an anti-Cry2Ab antibody (**b**). Gel filtration elution profiles of Cry2Ab (**c**) and Cry2Ab–AVM (**d**) on a Superdex 75 10/30 GL column: the homology modeling of Cry2Ab was shown at the top right corner of (**c**), the red area indicated the Lys 232 of Cry2Ab30 which was its potential bioconjugate site with 4”-O-succinyl avermectin; the schematic diagram of Cry2Ab–AVM was shown at the top right corner of (**d**), the red ring region showed 4”-O-succinyl avermectin was covalently conjugated onto Cry2Ab30. Fluorescence emission spectra of Cry2Ab30 (**e**), AVM (**f**), and Cry2Ab–AVM (**e**) using serial concentrations and 280 nm excitation.

Cry2Ab30 and Cry2Ab–AVM were detected by 10% SDS-PAGE with Coomassie brilliant blue staining (Figure 2a). Cry2Ab–AVM showed an obvious shift in molecular weight compared with Cry2Ab30 (65 kDa), which demonstrated that 4”-O-succinyl avermectin had been conjugated onto Cry2Ab30. Both Cry2Ab30 and Cry2Ab–AVM were detected by anti-Cry2Ab antibody (Figure 2b), and the results suggested that they were free of any major disturbances to the structure of Cry2Ab30 due to the bioconjugation of 4”-O-succinyl avermectin. We also ran size-exclusion chromatography to detect whether 4”-O-succinyl avermectin was conjugated onto the Cry2Ab30. As shown in Figure 2c,d, the elution time of Cry2Ab–AVM was earlier than Cry2Ab, suggesting that the bioconjugation of 4”-O-succinyl avermectin onto Cry2Ab30 increased the molecular weight of Cry2Ab30.

The fluorescence emission spectrum of Cry2Ab30 showed a main peak at 330 nm (Figure 2e). The fluorescence emission spectrum of AVM showed two peaks at 330 and 430 nm (Figure 2f). The main emission peak of AVM at 430 nm was also unique and distinguished it from Cry2Ab30. After coupling of 4”-O-succinyl avermectin onto Cry2Ab30, the fluorescence emission spectrum of Cry2Ab–AVM showed two peaks at 330 and 430 nm (Figure 2g), which suggested that 4”-O-succinyl avermectin was conjugated onto Cry2Ab30. Furthermore, an avermectin enzyme-linked immunosorbent assay using anti-avermectin antibodies could also detect AVM in Cry2Ab–AVM, but not in Cry2Ab30 (Table S1 and Figure S1), demonstrating that 4”-O-succinyl avermectin was conjugated onto Cry2Ab30.

These results suggested that Cry2Ab–AVM was successfully synthesized by bioconjugating 4”-O-succinyl avermectin onto Cry2Ab30.

### 2.2. Cry2Ab–AVM Binds to PxCR_10–11_ at Higher Affinity Than Cry2Ab30

**Figure 3:**
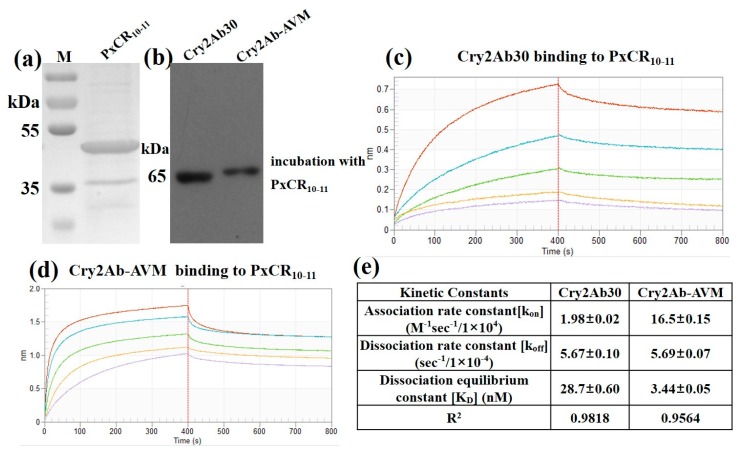
The binding of Cry2Ab30 or Cry2Ab–AVM to PxCR_10–11_. (**a**) The purity and identity of PxCR_10–11_ was evaluated by 10% SDS-PAGE. (**b**) Ligand blot detected the binding abilities of Cry2Ab30 and Cry2Ab–AVM to PxCR_10–11_. Binding kinetics of Cry2Ab30 (**c**) or Cry2Ab–AVM (**d**) to PxCR_10–11_ was studied by Fortebio biolayer interferometry, with Cry2Ab30 or Cry2Ab–AVM concentrations ranging from 38.5 to 576.9 nM. Kinetics were assessed by fitting data to a 1:1 binding model to determine the rate constants. (**e**) Summary of kinetic constants. Bioconjugation 4”-O-succinyl avermectin onto Cry2Ab30 could enhance the affinity of Cry2Ab30 to PxCR_10–11_.

In our previous study, PxCR_10–11_ is found to be the binding fragment of Cry2Ab30 in *P. xylostella* cadherin, and may be involved in the action of Cry2Ab30 against *P. xylostella* [23]. Qiu et al. also reported that the CR7-MPED region in the *S. exigua* cadherin played an important functional binding site for both Cry1Ac and Cry2Aa, but they did not compete for the same binding site, which suggested that they may bind to diverse cadherin protein epitopes. When cadherin transcription was suppressed by oral RNAi knockdown, the susceptibility of *S. exigua* larvae to both Cry1Ac and Cry2Aa was significantly reduced. These results suggested that cadherin serves as a specific Cry2A target [22]. Zhao et al. also reported that cadherin may function as receptors for Cry2Aa in *Helicoverpa armigera* and play important roles in the toxicity of the Cry2Aa toxin [29]. Here, PxCR_10–11_ was expressed in the *Escherichia coli* expression system and was further purified using a GST-Sefinose prepacked gravity column (Figure 3a). Ligand blot assay revealed that both Cry2Ab30 and Cry2Ab–AVM could bind PxCR_10–11_ (Figure 3b). To further assess the kinetic constants of Cry2Ab30 or Cry2Ab–AVM binding to PxCR_10–11,_ Fortebio biolayer interferometry was employed. As shown in Figure 3c,d, Cry2Ab30 and Cry2Ab–AVM both had strong binding affinity to PxCR_10–11_, characterized by rapid association and slow dissociation. Cry2Ab–AVM showed a significantly higher association rate constant (*k_on_*) than Cry2Ab30. The K_D_ value for the binding of Cry2Ab30 to PxCR_10–11_ was 28.7 nM, which was 8.34-fold larger than that of Cry2Ab–AVM (3.44 nM) (Figure 3e). The results revealed that bioconjugation engineering on Cry2Ab30 could significantly improve its binding affinity to PxCR_10–11_.

### 2.3. Insecticidal Toxicity Bioassay

**Figure 4:**
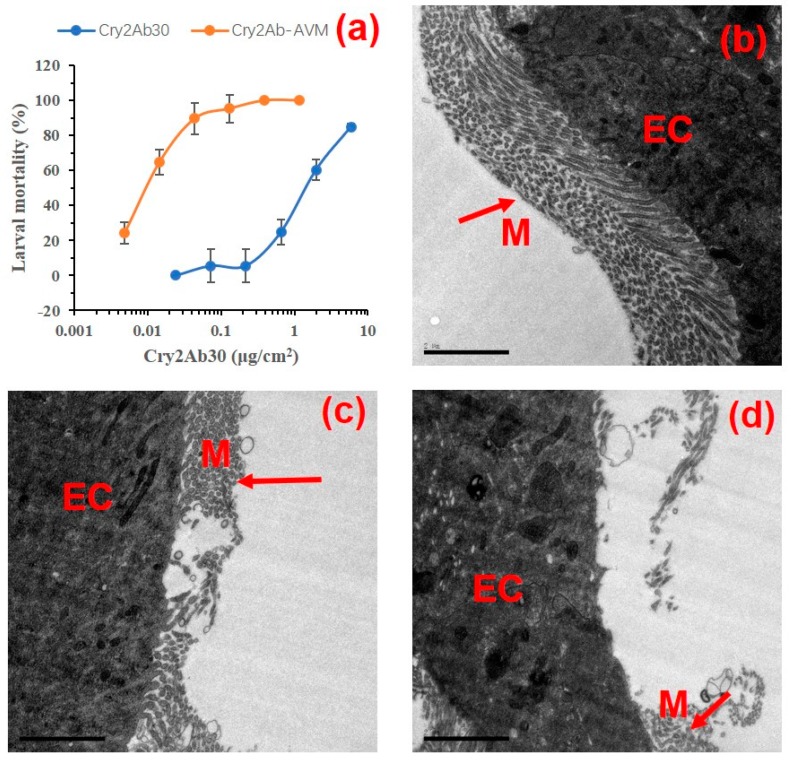
The insecticidal activities of Cry2Ab30 and Cry2Ab–AVM against *P. xylostella*. (**a**) The susceptibility of second-instar larvae of *P. xylostella* to Cry2Ab30 and Cry2Ab–AVM. Data are shown as the mean ± standard error of the mean (*n =* 3). Ultrastructural (TEM) imaging of general aspects of midgut of *P. xylostella* (**b**), the midgut fed with Cry2Ab30 (**c**), and midgut fed with Cry2Ab–AVM (**d**). EC = epithelial cell; M = microvilli. The scale bar is 2 μm.

We assayed the insecticidal toxicity of Cry2Ab30 or Cry2Ab–AVM against a susceptible laboratory population of *P. xylostella*. As expected, Cry2Ab–AVM exhibited substantially increased toxicity to *P. xylostella* larvae compared with Cry2Ab30 (Figure 4a), with median lethal concentration (LC_50_) values up to 154.4-fold lower than Cry2Ab30 (Table 1). The disruption of *P. xylostella* midgut epithelium fed by Cry2Ab30 or Cry2Ab–AVM was also assayed using transmission electron microscopy (TEM). In the control group, the *P. xylostella* midgut epithelial cells were intact, and the epithelial microvilli were neatly arranged (Figure 4b). The midgut cross sections showed extensive damage to *P. xylostella* midgut epithelium and epithelial microvilli that was induced by both Cry2Ab30 and Cry2Ab–AVM (Figure 4c,d). In addition, the degree of damage to the midgut epithelium was apparently greater in the Cry2Ab–AVM group relative to the Cry2Ab30 group, which was consistent with the higher toxicity of Cry2Ab–AVM to *P. xylostella*.

The insecticidal toxicity and TEM results together revealed that Cry2Ab–AVM showed a higher insecticidal potency than Cry2Ab30.

### 2.4. Modeling and Docking Analysis

**Figure 5:**
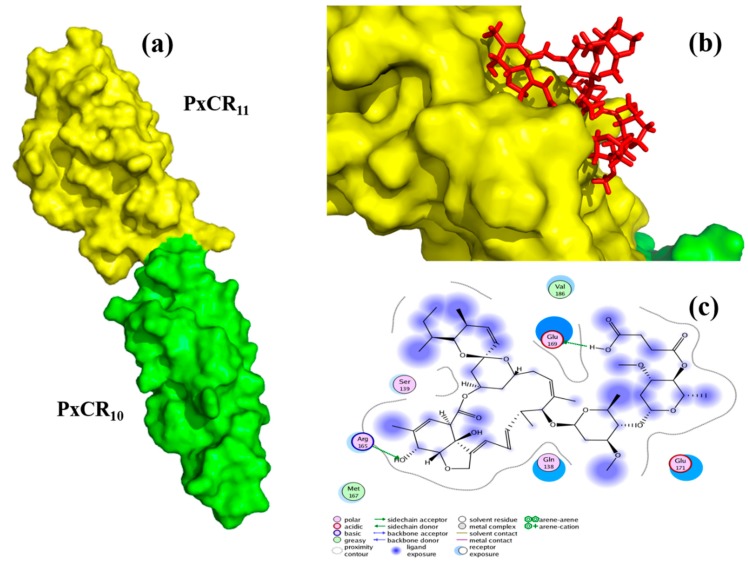
The interaction model of 4”-O-succinyl avermectin with PxCR_10–11_ by MOE docking study. (**a**) Surface diagram of PxCR_10–11_ by PyMol, PxCR_10_ is depicted as a green molecular surface. PxCR_11_ (membrane-proximal region) is depicted as a yellow molecular surface. (**b**) 4”-O-succinyl avermectin docked into the active site of PxCR_11_. 4”-O-succinyl avermectin is shown in the stick model in red. (**c**) Ligand interaction (planar projection of b) showed the possible interactions between 4”-O-succinyl avermectin and PxCR_10–11_. The solid blue ball of 4”-O-succinyl avermectin represents groups exposed to the PxCR_10–11_ surface.

We further investigated the interaction model of Cry2Ab–AVM ligand (4”-O-succinyl avermectin) with PxCR_10–11_ to explore the potential mechanism by which Cry2Ab–AVM binds to PxCR_10–11_ with higher affinity relative to Cry2Ab30 (Figure 5). The homology model of PxCR_10–11_ was firstly established by I-TASSER servers (Figure 5a). A Ramachandran plot, computed by the PROCHECK program, showed that there were 96.6% residues located on the allowed region in the PxCR_10–11_ model, suggesting that the model was reliable. We then predicted the docking sites using the site finder module implemented in Molecular Operating Environment (MOE). The predicted site was found close to the membrane-proximal region of PxCR_11_, which includes residues Asp 134, Thr 135, Gln 139, Glu 169, Phe 170, Glu 171, and Val 186. 4”-O-succinyl avermectin was then selected as the ligand against this predicted site using the docking module. The results suggested that the active carboxylic group of Cry2Ab–AVM ligand was exposed on the surface of PxCR_11_, which maybe covalently immobilized on Cry2Ab30. The macrolide lactone group of Cry2Ab–AVM ligand, after Cry2Ab–AVM bound to PxCR_10–11_, would dock into the activity site of PxCR_11_ (Figure 5b) and form a hydrogen bond with Arg 165 and other interactions with PxCR_11_ (Figure 5c). This may contribute to the elevated binding affinity of Cry2Ab–AVM with PxCR_10–11_ relative to Cry2Ab30.

## 3. Discussion

In this study, we engineered Cry2Ab30 to synthesize a Cry2Ab–AVM bioconjugate and enhanced the insecticidal potency against *P. xylostella* by up to 154.4-fold compared with Cry2Ab30. We tried to explore the potential mechanism for enhanced insecticidal activity of Cry2Ab–AVM. Protein binding is a crucial step in the insecticidal action of Cry1A [30,31]. It might also be a crucial step for the action for Cry2A as the two have a similar three-dimensional structure [32]. To our best knowledge, although numerous studies have attempted to identify the functional receptor of Cry2A, there no solid conclusions have been reached [33,34,35,36,37]. However, some papers suggested that cadherin is involved in the insecticidal action of Cry2A [22,29]. In our studies, we also found that both Cry2Ab30 and Cry2Ab–AVM could bind PxCR_10–11_. The results of the binding kinetics indicated that Cry2Ab–AVM bound PxCR_10–11_ at a higher affinity relative to Cry2Ab30, which may be a potential mechanism for the enhanced insecticidal activity of Cry2Ab–AVM. However, further study is required to understand whether this binding affinity is also consistent in the midgut brush border membrane of *P. xylostella* in vivo. In addition, there are other Cry2Ab30 binding proteins, such as PxAPN5 or soluble binding proteins in the midgut juice [38,39]. It is possible that bioconjugation engineering on Cry2Ab30 could also affect the affinity of CryAb30 with these binding proteins, but further study is required to evaluate this.

Avermectin is a 16-membered macrocyclic lactone derivative, with the macrolide lactone group in its active center [40]. We selected 4”-OH of avermectin to be carbonylated and to be conjugated onto Cry2Ab30, and the macrolide lactone of avermectin would be the functional ligand of Cry2Ab–AVM to interact with the binding proteins of Cry2Ab. The docking analysis suggested that the macrolide lactone group of Cry2Ab–AVM ligand docked into PxCR_10–11_ to form interaction forces which may enhance the binding affinity of Cry2Ab–AVM to PxCR_10–11_. To further verify the mechanism, 4”,5-O-succinyl avermectin was synthesized and conjugated onto Cry2Ab30 to synthesize Cry2Ab–AVMd according to the preparation of Cry2Ab–AVM. 4”,5-O-Succinyl avermectin has two carboxylic groups, each in either the 4” or 5 positions (Figure S2). The 5 position of avermectin was located in the macrolide lactone of avermectin and was more reactive than the 4” position [28]. The carboxylic group in the 5 position would be first conjugated onto Cry2Ab30 via EDC/NHS, which meant that the macrolide lactone of 4”,5-O-succinyl avermectin would not interact with PxCR_10–11_. As expected, the K_D_ value for the binding affinity of Cry2Ab–AVMd with PxCR_10–11_ was only 71.9 nM, which was almost the same as Cry2Ab30 (Table S2). In addition, the insecticidal toxicity of Cry2Ab–AVMd against *P. xylostella* was almost consistent with Cry2Ab30, with a relative potency of 1.06 (Table S3). These results suggested that the bioconjugation of avermectin onto Cry2Ab30 should be carried out in the 4” position of avermectin, which would swing the macrolide lactone group of avermectin to interact with the binding proteins of Cry2Ab30, which may be a key contributor in enhancing the affinity of Cry2Ab–AVM to its binding proteins. The bioconjugation strategy established here offers further scope for the engineering of Bt toxins for improved pest management.

## 4. Materials and Methods

### 4.1. Insects

A laboratory population of *P. xylostella* was supplied by Bio-Pesticide Engineering Research Center, Wuhan, China [41]. *P. xylostella* larvae were fed an artificial diet and were maintained under environmental conditions of 27 ± 2 °C, 70% humidity, and a 14 h/10 h (L/D) photoperiod.

### 4.2. Synthesis of 4”-O-Succinyl Avermectin

4”-O-Succinyl avermectin was synthesized according to previously reported procedures [42,43]. Selective protection of a hydroxyl group at the 5 position of avermectin (Aladdin, Shanghai, China) was carried out by *tert*-butyldimethylsilyl chloride (TBSCl) (Aladdin, Shanghai, China), and then succinylation of the 4”-hydroxy group was studied using succinic anhydride (Aladdin, Shanghai, China), followed by the deprotection of TBSCl by *p*-toluenesulfonic acid (PTSA) (Aladdin, Shanghai, China), thus synthesizing 4”-O-succinyl avermectin. ^1^H NMR (400 MHz, CDCl_3_) δ4.78 (d, *J* = 3.1 Hz, 1H), 4.68 (br, 2H), 3.36 (s, 3H), 2.69 (m, 4H), 1.87 (s, 3H). ^13^C NMR (101 MHz, CDCl_3_) δ 176.55, 173.73, 171.60, 139.51, 137.99, 137.85, 136.30, 135.17, 127.71, 124.77, 120.42, 118.33, 118.04, 98.33, 95.80, 94.99, 82.02, 80.71, 80.36, 79.23, 76.60, 75.61, 74.91, 68.45, 68.38, 67.68, 67.18, 66.45, 56.93, 56.54, 45.74, 40.45, 39.73, 36.51, 35.15, 35.00, 34.48, 34.22, 30.57, 29.07, 28.80, 27.49, 23.42, 20.69, 20.23, 19.90, 18.37, 17.32, 16.37, 15.10, 12.96, 12.04. HRMS (ESI): 995.4969 [M + Na]^+^.

### 4.3. Preparation of Cry2Ab30

The production and purification of Cry2Ab30 was as described in our previous study [20]. The plasmid of pET30a-*cry2Ab30* was transformed into *E. coli* BL21 (DE3) cells. The bacterial stab was inoculated onto the LB medium containing kanamycin (35 μg/mL) (Aladdin, Shanghai, China) and incubated at 20 °C. The expression of Cry2Ab30 was induced with 0.2 mM isopropyl-B-D-thiogalactopyranoside (IPTG) (Sangon, Shanghai, China) after the OD_600nm_ reached 0.6. After that, Cry2Ab30 was purified using a Ni-IDA prepacked column (Sangon, Shanghai, China).

### 4.4. Preparation of Truncated Recombinant P. xylostella Cadherin (PxCR_10–11_)

The 10th and 11th CR domains (1258–1602 aa) in the *P. xylostella* cadherin (PxCR_10–11_) binding Cry2Ab30 were expressed and purified as described in our previous study [23]. Cells were harvested by centrifugation, and the pellets were resuspended in glutathione transferase (GST) binding buffer (140 mM NaCl, 2.7 mM KCl, 10 mM Na_2_HPO_4_, 1.8 mM KH_2_PO_4_, pH 7.4). PxCR_10–11_ was purified using a GST prepacked gravity column (Sangon, Shanghai, China).

### 4.5. General Procedure for Cry2Ab–AVM

4”-O-Succinyl avermectin (2 × 10^−6^ mol) was firstly dissolved in 1 mL of DMF (Macklin, Shanghai, China). Then, triethylamine (2 × 10^−5^ mol) (Macklin, Shanghai, China) was added to provide protons, EDC (2 × 10^−5^ mol) (Aladdin, Shanghai, China) was added to form an O-acylisourea active intermediate, and NHS (5 × 10^−5^ mol) (Aladdin, Shanghai, China) was added to form an NHS ester intermediate; this intermediate was stirred for 0.5 h at 25 °C to activate 4”-O-succinyl avermectin. Cry2Ab30 (2 × 10^−8^ mol) was desalted into PBS (0.1 M, pH 7.4) using a size-exclusion column (GE Healthcare, Piscataway, NJ, USA), and activated 4”-O-succinyl avermectin was then added to Cry2Ab30 PBS buffer. The mixture was vortexed and allowed to react at room temperature for 2 h. After the reaction was completed, Cry2Ab–AVM was purified using a PD-10 desalting column (GE Healthcare, Piscataway, NJ, USA) [44]. Cry2Ab–AVM was detected by SDS-PAGE gel electrophoresis (Bio-Rad, Hercules, CA, USA) and size-exclusion chromatography using a Superdex 75 10/300 GL attached to an AKTA pure 150 (GE Healthcare, Uppsala, Sweden) at a flow rate of 0.8 mL/min [45]. The fluorescence spectra of Cry2Ab30 and Cry2Ab–AVM were recorded with a HORIBA Fluoromax-4 spectrofluorometer (HORIBA Scientific, Kyoto, Japan) at excitations of 280 nm (5 nm bandwidth) and 300–500 nm (5 nm bandwidth) emissions [46]. Furthermore, an avermectin enzyme-linked immunosorbent assay (Randox, Crumlin, UK) using anti-avermectin antibodies was also carried out to detect AVM in Cry2Ab–AVM according to the manual operation.

### 4.6. Ligand Blot Assay

The binding abilities of Cry2Ab30 and Cry2Ab–AVM to PxCR_10–11_ were detected by a ligand blot assay [47]. Cry2Ab30 and Cry2Ab–AVM were separated by 10% SDS-PAGE and transferred to a polyvinylidene fluoride (PVDF) membrane (Millipore, Darmstadt, Germany). The PVDF membrane was sealed with Tris-buffered saline Tween (TBST) buffer (20 mM Tris-HCl, 150 mM NaCl, 0.05% Tween-20, 0.1% BSA, pH 7.4) for 60 min with constant shaking. Then, the PVDF membrane was incubated with 10 ng/mL PxCR_10–11_ protein in TBST buffer overnight followed by three washes of TBST buffer.

### 4.7. Biosensor-Binding Kinetics

The binding kinetics of Cry2Ab30 or Cry2Ab–AVM to PxCR_10–11_ were assayed on an Octet Red 96 biolayer interferometer (Pall ForteBio Corp., Menlo Park, CA, USA) [48,49]. GST-tagged PxCR_10–11_ was first loaded onto anti-GST biosensors (Pall ForteBio Corp., Menlo Park, CA, USA) for 300 s. The binding of Cry2Ab30 and Cry2Ab–AVM to immobilized PxCR_10–11_ was measured with a 400 s association followed by a 400 s dissociation. The buffer was 10 mM GSH, 50 mM Tris, pH 8.0. The dissociation equilibrium constant (K_D_) was determined by Octet System Data Analysis software (Pall ForteBio Corp., Menlo Park, CA, USA) [50,51,52].

### 4.8. Toxicity Assays

The bioassays of Cry2Ab30 and Cry2Ab–AVM were performed with second-instar *P. xylostella*. Different doses of Cry2Ab30 and Cry2Ab–AVM solutions were spread on the surface of the diet and allowed to dry. Samples were treated with the same volume of PBS buffer as controls. Thirty individual larvae were used for each concentration. All the bioassay experiments were repeated in triplicates. Observations were recorded at 72 h, and the mortality was determined and statistically analyzed using SPSS 17.0 (Statistical Product and Service Solutions, Chicago, IL, USA) [53].

### 4.9. Transmission Electron Microscope Analysis

Third instar larvae of *P. xylostella* were starved for 5 h and then fed an artificial diet including Cry2Ab30 or Cry2Ab–AVM for 24 h. Larvae fed with PBS buffer (0.1 M, pH 7.4) were set as negative controls. The larvae were then dissected, and the midgut tissues were then isolated and immediately fixed in 2.5% glutaraldehyde (Aladdin, Shanghai, China) and post-fixed in 1% OsO_4_ (Aladdin, Shanghai, China). The fixed midgut tissues were then immersed into Epon for embedding. Ultrathin sections were sliced using a Leica EM UC7 ultramicrotome (Leica Microsystems, Wetzlar, Germany), and stained with uranyl acetate and then lead citrate [41]. The ultrastructure of midgut epithelium was examined using a transmission electron microscope (JEM-2100HC, JEOL, Tokyo, Japan).

### 4.10. Homology Modeling and Docking

The protein structure of PxCR_10–11_ was built by I-TASSER servers (http://zhanglab.ccmb.med.umich.edu/I-TASSER) and the molecular structure of 4”-O-succinyl avermectin was built using ChemBioDraw Ultra 12.0 (CambridgeSoft, Waltham, MA, USA). Docking of 4”-O-succinyl avermectin to PxCR_10–11_ was analyzed by Molecular Operating Environment (MOE) (Chemical Computing Group Inc., Montreal, QC, Canada) with default parameters; 4”-O-succinyl avermectin was selected as the ligand [54]. All structural images were rendered by PyMol (PyMol molecular graphics system, Schrödinger, LLC.) [55].

## Figures and Tables

**Table 1:** Bioassay of Cry2Ab30 and Cry2Ab–AVM against Susceptible Laboratory *Plutella xylostella* Larvae.

Toxin	LC_50_ (μg/cm^2^)	95% Confidence Interval	Slope	SE	Relative Potency ^a^
Cry2Ab30	1.544	1.041–2.402	1.922	0.333	1
Cry2Ab–AVM	0.010	0.006–0.016	1.792	0.356	154.4

^a^ Relative potency is normalized to the insecticidal activity (LC_50_ value) of Cry2Ab30.

## Supplementary Materials

The following are available online at https://www.mdpi.com/2072-6651/11/9/497/s1, Supplementary information includes details regarding the ELISA results of Cry2Ab–AVM, synthesis of 4″, 5-O-succinyl AVM, and the binding and toxicity assays of Cry2Ab–AVMd. Figure S1: The concentrations of AVM in series dilutions of Cry2Ab30 and Cry2Ab-AVM, Figure S2: The structural formula of 4″-O-succinoyl AVM and 4″, 5-O-succinoyl AVM, Table S1: The determination of avermectin (AVM) standards, Table S2: Kinetics of Cry2Ab-AVMd Binding to PxCR_10-11_ in the ForteBio System, Table S3: Bioassay of Cry2Ab30 and Cry2Ab-AVMd against Susceptible Laboratory *Plutella xylostella* Larvae.
