# Supplementary material for: Synthesis and Characterization of Cry2Ab–AVM Bioconjugate: Enhanced Affinity to Binding Proteins and Insecticidal Activity
Source: Toxins (Basel). 2019 Aug 27;11(9):497. doi: 10.3390/toxins11090497 (PMC6783867; doi:10.3390/toxins11090497)
Supplement: Supplementary file 1 [file toxins-11-00497-s001.pdf]

Supplementary Materials: Synthesis and characterization of Cry2Ab-AVM bioconjugate: enhanced affinity to binding proteins and insecticidal activity

Zhi-Zhen Pan, Lian Xu, Yi-Shu Zheng, Li-Yang Niu, Bo Liu, Nan-Yan Fu, Yan Shi, Qing-Xi Chen, Yu-Jing Zhu and Xiong Guan

ELISA analysis

Avermectins was detected by enzyme-linked immunosorbent assay (Randox, Crumlin, United Kingdom) according to the operation instruction. Briefly, avermectin antibody was firstly pre-coated on the microtitre plate. Avermectin, if present in the standard and sample competed with horseradish peroxidase labelled avermectin (enzyme labelled antigen) for a limited number of antibody sites on the microtitre plate. After incubation at room temperature to allow a competition reaction to take place, the microtitre plate was washed to remove excess reagents. The enzyme substrate was then added. After an incubation period to allow maximum color development, the color reaction was stopped by addition of acid. This produces a color change from blue to yellow, the color was inversely proportional to the concentration of avermectin. And the absorbances are read at 450 nm. A standard curve is then constructed to determine the avermectin concentration in the standard and sample by four parameter logistic regression (Table S1). The fitted equation was shown as below:

$$y = -0.033 + \frac{(3.877 + 0.033)}{1 + \left(\frac{x}{10.518}\right)^{0.943}}$$

Table S1. The determination of avermectin (AVM) standards.

Avermectins	concentration（ng/mL）	OD_{450nm}	CV[a]	B/B_0 (%)[b]
Standard 1	0	3.841	1.4	100
Standard 2	1.56	3.479	1.5	90.57537
Standard 3	3.51	2.6985	0.3	70.25514
Standard 4	7.9	2.1635	8.3	56.32648
Standard 5	17.78	1.55	1.9	40.35407
Standard 6	40	0.7855	9.3	20.4504

a CV (Coefficient of Variation) showed the extent of variability in relation to the mean of the tests. Each OD_{450nm} was test in two replicates. b B/B_0(%) has been normalized to the absorbance of standard 1 for each case.

Figure S1 showed that AVM was detected in Cry2Ab-AVM, but not in Cry2Ab30, demonstrating that AVM was coupled onto Cry2Ab30 (Figure S1).

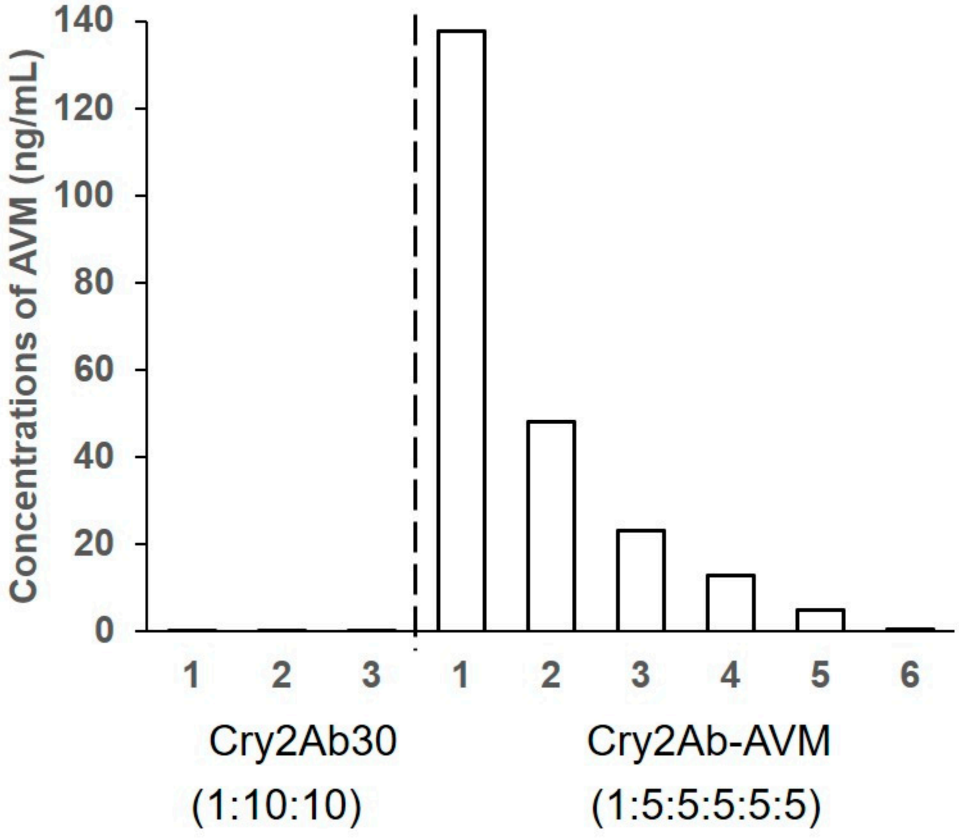

Figure S1. The concentrations of AVM in series dilutions of Cry2Ab30 and Cry2Ab-AVM. The maximum concentration of Cry2Ab30 and Cry2Ab-AVM is 37.1 and 16.6 mg/mL, respectively. The concentrations of AVM in series dilutions of Cry2Ab30 was not detected. But AVM could be detected in series dilutions of Cry2Ab-AVM which suggested that Cry2Ab-AVM was successfully synthesized by bioconjugation 4"-O-succinoyl AVM onto Cry2Ab30.

Synthesis of 4′′, 5-O-succinoyl AVM

4′′, 5-O-succinoyl avermectin was synthesized according to previously reported procedure (Fig. S2). ^{1}H NMR (400 MHz, $CDCl_3$) δ 4.80(s,1H), 4.09(d,J=6.3Hz,1H), 3.38(s,3H), 2.27-2.75(m,4H), 2.66-2.70(m,4H), 1.17(s,3H). ^{13}C NMR (101 MHz, $CDCl_3$) δ 177.26 , 177.14 , 173.51 , 171.89 , 171.52 , 139.11 , 137.96 , 135.25 , 135.06 , 133.31 , 125.03 , 124.76 , 120.88 , 120.39 , 118.38, 98.40 , 97.55 , 94.89 , 81.92 , 80.84 , 80.51 , 79.26 , 75.63 , 70.44, 68.97, 68.21 , 67.25, 67.17 , 66.46 , 56.90, 56.55 , 45.72 , 41.19 , 39.70 , 36.88, 35.52 , 35.47 , 35.01 , 34.52, 34.06 , 32.20 , 31.91 , 31.44 , 31.22 , 30.21, 29.68 , 29.35 , 29.04 , 28.92 , 28.83, 28.74, 27.29 , 26.39 , 23.42 , 22.68 , 20.26 , 19.44, 18.38 , 17.45 , 17.32 , 15.16, 14.11 , 12.43 , 12.01. ESI-MS (m/ z):1095.45[M +Na]$^{+}$

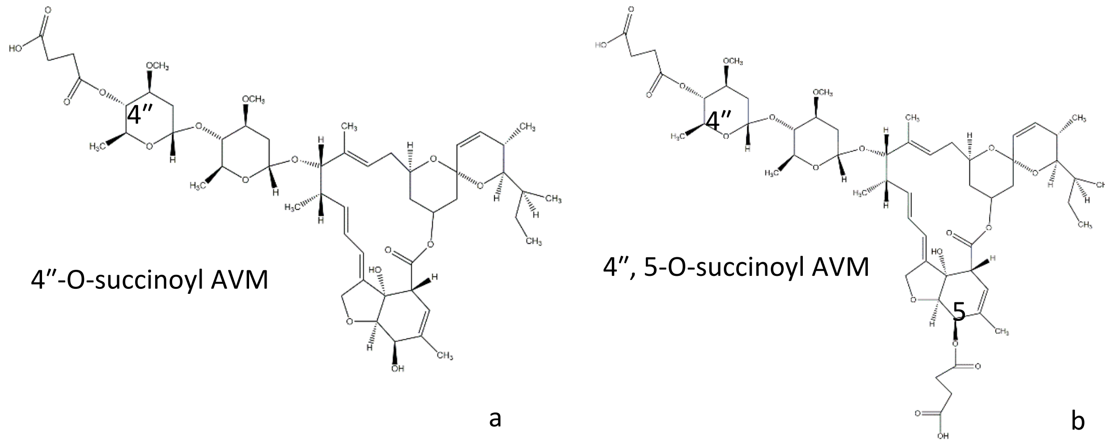

Figure S2. The structural formula of 4″-O-succinoyl AVM (**a**) and 4″, 5-O-succinoyl AVM (**b**).

The general procedure for Cry2Ab-AVMd (4″, 5-O-succinoyl AVM was conjugated with Cry2Ab30 via EDC/NHS) was the same as Cry2Ab-AVM. The binding assays and toxicity assays of Cry2Ab-AVMd were also same as Cry2Ab-AVM. The K_D value for the binding of Cry2Ab-AVMd to $PxCR_{10-11}$ was 71.9 nM, which was almost the same as Cry2Ab30. The bioconjugation of Cry2Ab30 with Cry2Ab-AVMd would not improve its affinity to $PxCR_{10-11}$ (Table S3). The insecticidal toxicity of Cry2Ab-AVMd was also the same with Cry2Ab30 against *P. xylostella,* the relative potency was 1.06 in compared with to Cry2Ab30 (Table S4).

Table S2. Kinetics of Cry2Ab-AVMd Binding to $PxCR_{10-11}$ in the ForteBio System.

	**K_D (M)**	**K_D Error**	**Kon (1/Ms)**	**Kon Error**	**K_{off} (1/s)**	**K_{off} Error**	**R^2**
Cry2Ab-AVMd	7.19E-08	2.43E-09	1.18E+05	3.81E+03	8.51E-03	8.71E-05	0.9443

Table S3. Bioassay of Cry2Ab30 and Cry2Ab-AVMd against Susceptible Laboratory *Plutella xylostella* Larvae.

**Toxin**	**LC_{50}（μg/cm^2）**	**95% confidence interval**	**Slope**	**SE**	**Relative potency [a]**
Cry2Ab30	1.544	1.041 - 2.402	1.922	0.333	1
Cry2Ab-AVMd	1.455	0.742-3.623	0.853	0.165	1.06

a Relative potency is normalized to the insecticidal activity (LC50 value) of Cry2Ab30.
